# Clinical Features of COVID‐19 Associated Pulmonary Aspergillosis: A Multicenter, Retrospective Study

**DOI:** 10.1111/crj.70048

**Published:** 2025-01-26

**Authors:** Yasheng Zhan, Guojun He, Cheng Zhong, Yake Yao, Jiangying Zhou, Tong Li, Hua Zhou

**Affiliations:** ^1^ Department of Respiratory and Critical Care Medicine, The First Affiliated Hospital Zhejiang University School of Medicine Hangzhou Zhejiang China; ^2^ Department of Critical Care Medicine Jinhua People's Hospital Jinhua Zhejiang China; ^3^ Department of Critical Care Medicine, The First Affiliated Hospital Zhejiang University School of Medicine Hangzhou Zhejiang China; ^4^ Department of Respiratory and Critical Care Medicine, Affiliated Hangzhou Chest Hospital Zhejiang University School of Medicine Hangzhou Zhejiang China

**Keywords:** CAPA, COVID‐19, ICU, invasive pulmonary aspergillosis

## Abstract

**Objective:**

This study was conducted to further understand the clinical characteristics of COVID‐19 associated pulmonary aspergillosis (CAPA).

**Methods:**

In this study, we conducted a multicenter retrospective survey, which included patients with COVID‐19 from five hospitals in Zhejiang, China. A total of 197 patients with COVID‐19 were included in the study. The detailed clinical data of seven patients with CAPA from COVID‐19 onset to 28 days after CAPA were collected and analyzed.

**Results:**

In the total of 197 patients, 36 were admitted to the intensive care unit (ICU), 13 received mechanical ventilation; among them, nine received extracorporeal membrane oxygenation (ECMO). All seven cases acquired CAPA in the ICU, six cases during MV, of which five cases received ECMO treatment at the same time, and one case had been off ventilation. The average duration from onset of COVID‐19 to CAPA was 25.4 days, from ICU admission to CAPA was 23.4 days, and from MV to CAPA was 22.1 days. All seven patients were diagnosed with CAPA without neutropenia, four with lymphopenia, seven with decreased CD4+ T lymphocyte, and five with decreased CD8+ T lymphocyte. All cases received glucocorticoids before CAPA, with an average duration of 15 days and an average cumulative dose of 762.5 mg prednisolone. In addition, all patients suffered bacterial infections and received antibacterial agents before CAPA, with an average duration of 22.6 days. CAPA was diagnosed according to a positive culture of *Aspergillus fumigatus* in sputum or bronchoalveolar lavage fluid (BALF) and positive serum 1,3‐β‐d‐glucan in all seven patients; serum galactomannan was positive in three cases. Rhizopus was cultured from BALF of one case during treatment of CAPA. All patients received antifungal therapy, and the 28‐day survival rate was 100%.

**Conclusion:**

The incidence of CAPA in patients with COVID‐19 admitted to the ICU was 19.44%, all patients with CAPA had a history of chronic underlying diseases, and all had a history of high dose glucocorticoid. Patients with CAPA had no specific clinical symptoms and lung imaging manifestations, and diagnosis depended on Aspergillus culture and galactomannan detection. For patients with COVID‐19 with these high‐risk factors, *Aspergillus* culture and GM testing should be performed actively to avoid delaying the diagnosis of CAPA.

## Graphical abstract

In the total of 197 patients, seven cases acquired CAPA. The average duration from onset of COVID‐19 to CAPA was 25.4 days. All cases received glucocorticoids before CAPA, with an average duration of 15 days and an average cumulative dose of 762.5 mg prednisolone. All patients received antifungal therapy, and the 28‐day survival rate was 100%.

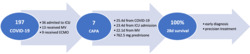

## 1 Introduction

Sars‐Cov‐2 is a strain that causes COVID‐19 and mainly infects the respiratory tract. Although most infected people develop mild to moderate disease and recover without hospitalization, some patients may develop severe pneumonia [1]. Respiratory failure and septic shock are the most severe symptoms. The infection usually leads to impaired systemic and airway local immune function, such as the decline of macrophage function, low leukocyte, and the destruction of airway mucosal integrity, which may occur secondary to bacterial or fungal infection [2, 3]. Critically ill patients with COVID‐19 are also prone to excessive immune activation and inflammatory factor storm [4]. These patients are usually treated with glucocorticoids [5], which further inhibit leukocyte traffic and thereby the access of leukocytes to the site of inflammation [6]. Moreover, critically ill patients frequently develop infections, thus requiring long‐term broad‐spectrum antibiotics.

Invasive pulmonary aspergillosis (IPA) secondary to influenza virus pneumonia has been increasingly reported over the recent years. IPA has been also reported in patients with severe COVID‐19. *Aspergillus* is the most important fungus causing invasive pulmonary infection [7], especially in immunocompromised hosts. Typical risk factors of invasive pulmonary aspergillosis (IPA) are agranulocytosis, hematopoietic stem cell transplantation, solid organ transplantation, long‐term use of glucocorticoids, and chronic obstructive pulmonary disease [8, 9]. Moreover, many studies suggested that severe influenza pneumonia is another risk factor for IPA [10]. Studies reported that the incidence of IPA in patients with COVID‐19 undergoing mechanical ventilation fluctuates from 2.4% to 35% [11, 12]. We conducted a multicenter retrospective survey, which included 197 patients with COVID‐19 across five hospitals in Zhejiang, China, to understand the clinical characteristics of COVID‐19 associated pulmonary aspergillosis (CAPA).

## 2 Methods

### 2.1 Study Design and Participants

This retrospective, multicenter cohort study included five hospitals in Zhejiang Province, i.e., The First Affiliated Hospital, School of Medicine, Zhejiang University, The First Hospital of Jiaxing, Taizhou Hospital of Zhejiang Province, The First people's Hospital of Yuhang District of Hangzhou City, and The Deqing People's Hospital. All hospitals were designated hospitals for COVID‐19 in Zhejiang Province. All adult patients with COVID‐19 admitted by May 18, 2020, were included in the study. COVID‐19 cases were confirmed based on the WHO interim guidelines. Only patients with a laboratory‐confirmed infection were enrolled in the study.

### 2.2 Data Collection

Demographic, clinical features, laboratory examination, viral nucleic acid detection, pulmonary imaging, microbiology data, and other data were obtained through patient medical records. Medical staff treating patients with COVID‐19 was responsible for collecting and reviewing the data. We used a standardized case report form to collect clinical data. Data were verified by two physicians, while disagreements were resolved by inviting a third researcher (HZ). Concerning ambiguous data, study investigators in Hangzhou contacted the doctor responsible for treating the patient for clarification. The deadline for data collection was May 18, 2020.

### 2.3 Laboratory Tests, X‐Ray, and Lung Computed Tomography Scan (CT)

Laboratory tests included a complete blood count, coagulation profile, CRP, G test, GM test, levels of interleukin‐6 (IL‐6), etc. In addition, X‐ray and chest CT scans were performed for all inpatients. The treating physician determined the frequency and type of test based on clinical symptoms.

### 2.4 Tracheoscopy and Microbiological Examination

Patients without endotracheal intubation did not undergo tracheoscopy and alveolar lavage. Bacterial and fungal cultures were performed using sputum samples. Patients with endotracheal intubation underwent tracheoscopy according to clinical needs. The main purpose was to clean respiratory secretions, while the obtained bronchial aspirates or alveolar lavage fluid were routinely smeared and cultured with bacteria and fungi.

### 2.5 Definitions

The diagnostic criteria of COVID‐19 were clinical symptoms of respiratory tract infection and positive detection of Sars‐Cov‐2 virus nucleic acid in respiratory tract specimens (nasopharyngeal swabs or sputum). Patients were defined as severe cases when one of the following criteria were met: arterial oxygen pressure (Pa02)/fraction of inspired oxygen (Fi02) ≤ 300 mm Hg (1 mm Hg = 0.133 kPa), oxygen saturation ≤ 93% at resting state, or respiratory rate ≥ 30/min. Patients who met any of the following criteria were defined as critically ill: respiratory failure and requirement for mechanical ventilation, shock, in combination with other organ failure requiring ICU care. The definition used to diagnose IPA was based on the presence of clinical, radiological, and mycological criteria in all patients with invasive pulmonary aspergillosis (panel) [13]. Every COVID‐19 patient was reviewed, and a consensus was achieved to ascertain whether the invasive pulmonary aspergillosis definition was met.

### 2.6 Ethics and Informed Consent

This retrospective study was approved by the medical ethics committee of the First Affiliated Hospital Zhejiang University School of Medicine and exempt from informed consent; ethics approval no. IIT20210578A. The requirement for informed consent was waived by the Ethics Commission due to the retrospective and anonymous characters of the study. We confirmed that the data was anonymized and maintained with confidentiality, compliance with the Declaration of Helsinki.

## 3 Results

A total of 197 patients with COVID‐19 were included in the study. There were four cases with a mild type, 93 cases with a common type, 71 cases with a severe type, and 29 critically ill patients at the time of admission. In the course of disease development, 36 patients were treated in ICU, 13 of whom received endotracheal intubation and mechanical ventilation. Among them, nine received ECMO. The data collection time of this study was as of May 18, 2020. All patients with Sars‐Cov‐2 virus nucleic acid had turned negative, discharged, or transferred out of the isolation ward for basic disease treatment, and all patients with CAPA had been diagnosed for 28 days. Eight patients were positive for *Aspergillus* culture in airway secretion during the study period. According to IPA clinical diagnostic standard [13], one patient was diagnosed based on airway colonization, and seven patients were determined as clinical diagnostic cases. The clinical characteristics of seven patients with CAPA were analyzed.

### 3.1 Clinical Characteristics of Seven Patients When COVID‐19 was Diagnosed

All seven patients were male, with an average age of 74 years (59–90 years). They all acquired Sars‐Cov‐2 infection in the community. There were six severe cases and one critically ill patient at the time of diagnosis (COVID‐19). Two patients had a history of smoking. Seven patients had complications, including chronic obstructive pulmonary disease (COPD) and chronic renal failure. The main clinical manifestations were fever, cough, and similar (Table 1).

**TABLE 1:** Characteristics of patients on admission.

CASE	Age (year)	Coexisting conditions	Smoking history	Symptom	Time from illness onset (day)	Severity^a^
1	90	COPD, HTA	60 pack‐years	Fever, fatigue	3	Severe
2	59	Liver transplantation status	No	Fever, cough, sputum, muscle aches	4	Severe
3	74	DM, HTA, previous inactive pulmonary tuberculosis	No	Cough, dizziness, runny nose, fever	8	Critical
4	84	COPD, HTA, CHD	No	Fever	2	Severe
5	62	HTA	No	Fever, cough	1	Severe
6	61	DM, HTA, CRF, HD	20 pack‐years	Cough and cough sputum	1	Severe
7	90	AF, HTA, CHD, CKD	No	Fever, cough, shortness of breath	1	Severe

Abbreviations: AF, atrial fibrillation; CHD, coronary atherosclerotic heart disease; CKD, chronic kidney disease; COPD, chronic obstructive pulmonary disease; CRF, chronic renal failure; DM, diabetes mellitus; HD, hemodialysis; HTA, hypertension.

^a^ According to the diagnosis and treatment plan for novel coronavirus pneumonia (trial 7^th^ edition), it is divided into mild/ordinary type and severe/critical type.

### 3.2 Clinical Features in the Diagnosis of CAPA

Seven patients were critically ill at the time of diagnosis of CAPA, and all had a history of endotracheal intubation and mechanical ventilation (MV). At the time of diagnosis, one patient was weaned from the ventilator and received nasal catheter oxygen (oxygen flow 2 L/min), while six were still undergoing MV, five of whom were simultaneously on VV‐ECMO therapy. Among six patients who required bedside CRRT, five had an acute renal impairment, and one had a history of uremia. The APACHEII score of seven patients averaged 24.1 (16.0–31.0). The average duration from diagnosis of COVID‐19 to CAPA was 25.4 days (11–36 days); the average duration from admission to ICU to CAPA was 23.4 days (9–49 days), and the average time from MV to CAPA was 22.1 days. All the seven cases received glucocorticoids before CAPA, with an average duration of 15 days and an average cumulative dose of 762.5 mg (50.8 mg/day) Figure 2, Table 2.

**TABLE 2:** Clinical features in the diagnosis of CAPA.

CASE	Respiratory support modalities	ARF	CRRT	APACHE II Score	Duration of COVID‐19 before CAPA (days)	ICU stay before CAPA (days)	MV before CAPA (days)	Total dose of glucocorticoids before CAPA (mg)
1	ECMO + MV	Yes	Yes	16	12	11	4	625
2	ECMO + MV	Yes	Yes	28	29	13	11	1050
3	ECMO + MV	Yes	Yes	27	29	29	29	838
4	ECMO + MV	No	No	31	11	9	9	650
5	ECMO + MV	Yes	Yes	21	36	49	49	750
6	nasal catheter oxygen inhalation	No	Yes	26	32	28	28	1175
7	MV	Yes	Yes	20	29	25	25	250

Abbreviations: APACHEII score, acute physiological score, age score, and chronic health score; ARF, acute renal failure; CAPA, COVID‐19 associated pulmonary aspergillosis; CRRT, continuous renal replacement therapy; ECMO, extracorporeal membrane oxygenation; MV, mechanical ventilation.

### 3.3 Features of Laboratory Tests in the Diagnosis of CAPA

Only one out of the seven patients had a decreased white blood cell count, and four patients had a low lymphocyte count. Seven patients had decreased CD^4+^, and five patients had decreased CD^8+^. IL‐6 and IL‐10 of all seven patients and CRP of six patients were elevated. Serum 1, 3‐β‐d‐glucan of all seven patients was elevated, with an average of 96.7 pg/mL (range 61.4–115.2 pg/mL). When CAPA was confirmed, serum galactomannan was detected in six patients and was elevated in three cases, with the average of 1.1 pg/mL Table 3.

**TABLE 3:** Laboratory results of patients in the diagnosis of CAPA.

Case	1	2	3	4	5	6	7
Leukocyte count (4–10.0 × 10^9^/L)	15.9	4.2	6.4	10.6	16.2	3.5	5.3
Lymphocyte count (0.8–4.0 × 10^9^/L)	0.2	0.1	0.3	1	2.5	0.4	1.1
Neutrophil count (2–7.0 × 10^9^/L)	15.3	4	5.5	10.2	7.3	3	2.8
CD4^+^ T lymphocyte (550–1440/μL)	206	13	119	21	323	159	537
CD8 + T lymphocyte (320–1250/μL)	181	22	58	17	783	113	417
Platelet count (83–303 × 10^9^/L)	124	33	31	103	69	123	158
Hemoglobin (131–172 g/L)	104	97	89	108	79	81	95
C‐reactive protein (0.0–8.0 mg/L)	22.85	116.67	182.32	5.07	15.64	204.03	29.84
Interleukin‐6 (0–6.61 pg/mL)	11.79	66.43	883.89	57.18	110.75	28.55	102.94
Interleukin‐10 (0–2.31 pg/m)	2.72	14.9	15.9	4.58	10.6	<28.55	6.22
Albumin (40.0–55.0 g/L)	36	33.2	29.4	30.7	37.8	40.1	43.2
Serum 1, 3‐β‐d‐glucan (< 60 pg/mL)	61.4	115.2	176	78.2	67.4	80.3	98.6
Serum Galactomannan (< 0.5/L)	NA	0.17	4.8	0.53	0.54	0.16	0.45

### 3.4 Microbiological Characteristics in Patients With CAPA

**FIGURE 1:**
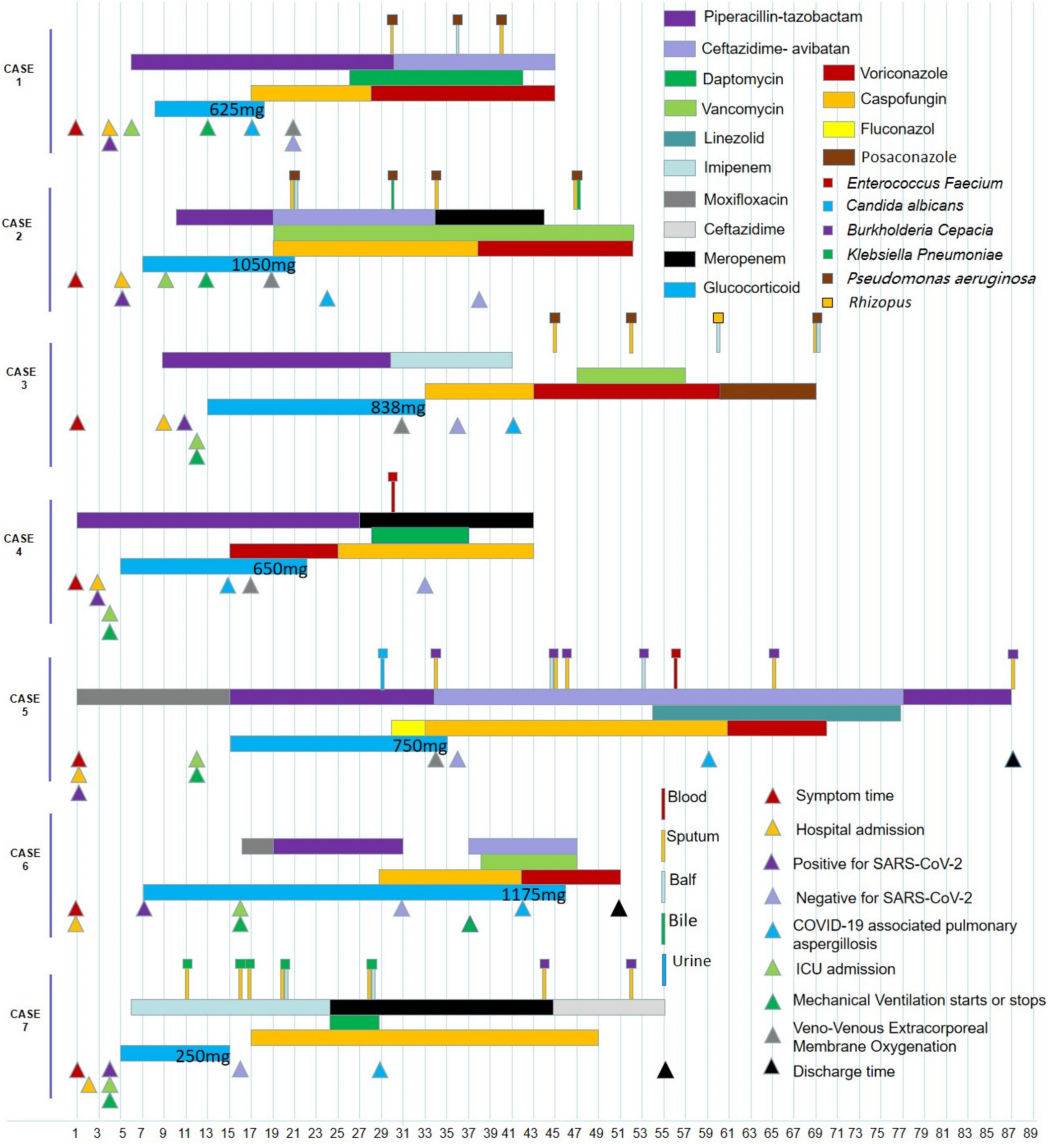
The courses of the disease in seven patients with CAPA. The doses of glucocorticoids were cumulative doses of prednisone.

*Aspergillus fumigatus* was found in all seven patients with CAPA (one sputum sample and six alveolar lavage fluid samples). *Rhizopus* was found in bronchoalveolar lavage fluid of one case. At the time of CAPA, six patients were complicated with bacterial pneumonia, including three cases of *Pseudomonas aeruginosa* , two cases *Burkholderia cepacia* , one case of *Klebsiella pneumoniae* ; two patients were complicated with *Enterococcus faecalis* bloodstream infection; one patient had urinary tract infection caused by *Candida albicans* . All seven patients had a history of antibacterial drug use before CAPA, with an average duration of 22.6 days. Five patients had a history of caspofungin Figure 1.

### 3.5 Imaging Features

**FIGURE 2:**
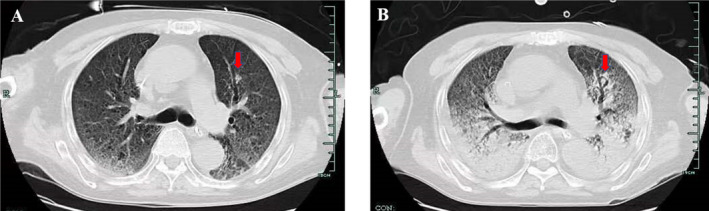
Lung CT findings of case 1. (A) The CT image was taken one day after the diagnosis of CAPA. It is showing diffuse ground glass shadow, interstitial lesions in both lungs and the small nodule in the left upper lung. (B) A follow‐up of CT after 1‐week showed that consolidation was aggravated; there was a cavity and crescent sign in the upper left.

All seven patients had a wide range of lesions that involved all five pulmonary lobes as determined by lung CT. Pulmonary CT findings included diffuse ground glass shadow, consolidation, cavity, nodule, and pleural effusion. Pulmonary CT did not show typical images of *Aspergillus* infection due to diffuse pulmonary lesions and fibrosis caused by Sars‐Cov‐2 infection Figure 2.

### 3.6 Treatment and Prognosis of Patients With CAPA

All patients received antifungal therapy after CAPA, which included voriconazole, caspofungin, or posaconazole. Two patients were nebulized with amphotericin B. All seven patients survived 28 days after CAPA Figure 1.

## 4 Discussion

In this study, the incidence of CAPA in patients with COVID‐19 admitted to the ICU was 19.44% (7/36), which was similar to the results of two single‐center retrospective studies on CAPA (20.6% (7/34) [14] and 19.6% (6/31) [15]). The situation of secondary IPA after Viral pneumonia (such as influenza) may vary between institutions and within an institution over time [16]. CAPA occurred in patients with respiratory failure due to SARS‐Cov‐2 pneumonia. It is believed that severe viral pneumonia leads to pulmonary epithelial damage and immune dysfunction that makes patients more susceptible to invasive pulmonary aspergillosis [17]. A previous study reported that severely ill patients in ICU are at high risk of developing IPA [14]. Similarly, studies have reported a positive correlation between a higher disease severity score (APACHEII score) and IPA [18, 19]. In addition, chronic obstructive pulmonary disease and previous inactive pulmonary tuberculosis are risk factors of *Aspergillus* infection [20]. In this study, all patients with CAPA had a history of chronic underlying diseases.

Patients in this study were treated with high cumulative doses of glucocorticoids and broad‐spectrum antimicrobials. Studies have shown that the use of glucocorticoids [15], lung injury during COVID‐19, and use of broad‐spectrum antibiotics are important risk factors for CAPA, similar to influenza‐secondary IPA [18]. Moreover, a multicenter, multi‐country study found that invasive mechanical ventilation and advanced age were significantly associated with CAPA infection; yet, no significant association between systemic glucocorticoid use and CAPA was observed [21]. Thus, the association between glucocorticoid use and CAPA needs to be further explored. Unfortunately, due to the low sample size, corticosteroid dosage and time of use and the correlation between CAPA occurrence were not thoroughly investigated in this study.

In this study, seven patients were diagnosed with CAPA. Patients were elderly men with non‐neutropenia, which is consistent with the findings of Kosmidis et al. [22].

Activated CD4+ and CD8+ T lymphocytes can help clear acute infection and prevent protective immunity by establishing immune memory to prevent reinfection [23]. Gao et al. reported a significant reduction in CD4+ T cells and CD8+ T cells in patients with acute COVID‐19 pneumonia [24]. In this study, CD4+ T cells in all patients with CAPA and CD8+ T cells in 71.43% cases were decreased. Thus, the lack of protective immunity might also be one of the factors leading to CAPA.

Seven patients had elevated IL‐6 and IL‐10. These pro‐inflammatory cytokines are commonly elevated in patients with severe COVID‐19. IL‐6 is a multifunctional cytokine that has an important role in protective immunity against *Aspergillus*. A significant increase in IL‐6 after infection with *A. fumigatus* can affect immune cell function, and immune cell antiviral ability [25]. It may also lead to the complications of fatal COVID‐19 [26, 27]. The above findings are consistent with elevated serum IL‐6 and IL‐10 levels in patients with pandemic influenza (H1N1). Similarly, patients with respiratory viral infections with elevated IL‐10 are more susceptible to IPA [28].

Three out of the seven patients with CAPA had a positive serum GM test. Since the seven patients were non‐neutropenia patients, the sensitivity of the GM test in alveolar lavage fluid was higher than serum [9, 29]. However, during the COVID‐19 epidemic, all patients underwent only serum GM tests due to the limitation of materials, medical, and other resources. *A. fumigatus* was found in all seven patients with CAPA. *A. fumigatus* has been reported as a very common genus of *Aspergillus* co‐infection with COVID‐19. Based on culture results, all patients in this study underwent antifungal therapy; 85.72% of patients were treated with voriconazole or caspofungin, which are the most common antifungal drugs. In addition, one patient developed *Rhizopus* infection during CAPA antifungal therapy and adjusted the antibiotic to posaconazole. All patients survived at 28 days under antifungal treatment.

In this study, all patients with CAPA had a history of chronic underlying diseases and high dose glucocorticoid, had no specific clinical symptoms and lung imaging manifestations. So, for patients with COVID‐19 or other severe viral pneumonia (such as influenza), if they have high risk factors of Aspergillus infection, Aspergillus culture and GM testing should be performed actively to avoid delaying the diagnosis of IPA.

Our study has following limitations: a small sample size; no in‐depth exploration was performed on other influencing factors. Thus, larger studies with more clinical information are required to confirm these findings.

## Author Contributions

All authors made a significant contribution to the work reported, whether that is in the conception, study design, execution, acquisition of data, analysis and interpretation, or in all these areas; took part in drafting, revising or critically reviewing the article; gave final approval of the version to be published; have agreed on the journal to which the article has been submitted; and agree to be accountable for all aspects of the work.

## Conflicts of Interest

The authors declare no conflicts of interest.

## Data Availability

The data that support the findings of this study are available from the corresponding author upon reasonable request.
